# Are theoretical results ‘Results’?

**DOI:** 10.7554/eLife.40018

**Published:** 2018-07-23

**Authors:** Raymond E Goldstein

**Affiliations:** 1 Department of Applied Mathematics and Theoretical Physics, Centre for Mathematical SciencesUniversity of CambridgeCambridgeUnited Kingdom

**Keywords:** theory, mathematical modeling, scientific publishing, interdisciplinary research, physics

## Abstract

Yes.

## Introduction

The decision letter from the journal was very supportive – it was clear our paper (Kirkegaard et al., 2016) would be published – but one of the referees definitely did not like the way we had combined experimental biology and physical calculations in our paper: “The data should be described and the inferences drawn, and the modelling relegated to its proper place as quantitative verification of the inferences that can be made directly from the data.”

And this was not an isolated case; a referee of another paper had said: “Instead, the authors should let the data speak for itself, and postpone heavier theoretical analysis for later, perhaps in the Discussion.” Many of my colleagues have experienced the same reaction to papers mixing theory and experiment. What were we doing wrong? Why was it not OK, according to these referees, to present the observations and the theory in a back-and-forth dialogue within the ‘Results’ section?

While I was bemused by these statements (relegated!), they resonated with my long experience with some in the biology community, namely that they see the significance of theory very differently from the way physicists understand it. For many biologists, theoretical results are simply not ‘Results’. Indeed, I suspect to many they are seen as a matter of opinion, without any intrinsic significance. In essence, they don’t add anything new. Hence the belief in the canonical Results/Discussion dichotomy in which theory (or ‘modelling’, as it is often called) plays second fiddle, or third.

In contrast, physicists are brought up to think by means of mathematical models: harmonic oscillators, random walks, idealized electrical circuits and so on are among the tools in our toolbox, whether we do experiment or theory. We use them as solvable examples in which a well-defined set of assumptions leads to precise outcomes, and where the dependence of the outcomes on the various parameters in the model can be interpreted. This approach allows us to estimate what is important and what is not in any setting. Models also help us to think about problems: “If this is the underlying physics, then A should vary with B quadratically…”, or “under these assumptions, the data should collapse like this…” or, when we spot something is not quite right, “here I argue that these claims are in conflict with basic laws of physics” (Meister, 2016).

The role of theory is also intimately connected with *predictions*. While I know biologists who would say “who cares about a prediction in the absence of experiment?”, physicists are brought up to celebrate them – they are the stuff of legend, from Dirac’s prediction of antiparticles and Einstein’s prediction of the bending of starlight, to the work by many that predicted the Higgs particle. We view predictions as motivations for experiment and as a means to move the discipline forward. Of course, sometimes they turn out to be wrong, but that is often how science works. Even if theoretical work does not take the form of a prediction, *per se*, it may still be very useful to design experiments with theory in mind, as emphasized by Bialek (2018), who has described many historical examples of the role theory has played in biology, from Rayleigh’s work on hearing to Watson and Crick.

My purpose here is to push back against the view that theory is not a ‘Result’. I argue for the unabashed inclusion of mathematical formulations and pedagogy within the body of papers published in *eLife* and other primarily biological journals. By interleaving the experimental and theoretical results it is possible to tell a story, and I firmly believe this makes for much more interesting and readable papers. It is also faithful to the scientific method, in which one goes back and forth with experiment and hypothesis.

Readers may be interested to learn that biological information, background and results are now routinely included in papers published in physics journals, although this has not always been the case: I vividly recall a situation several decades ago when a colleague, a high-energy physicist, saw a preprint about pattern formation in the slime mold *Dictyostelium discoideum* on my desk and asked: “Why would any physicist study something as ridiculous as that?” But by now many physicists do exactly that, and many physics journals are full of discussions of cAMP signaling, spiral waves, and chemotaxis (Goldstein, 1996; Rappel et al., 1999; Gholami et al., 2015). If we really take interdisciplinary research seriously then I assert there has to be a prominent place for theory within biology papers, both as Results in papers that combine experiment and theory, and as Results in theory papers.

This is nothing new. If you have not already done so, I highly recommend reading the celebrated paper by Hodgkin and Huxley (1952) to see experiments and theory interleaved. Theory is not relegated to the discussion, or worse, to supplementary material, but instead is incorporated into the body of the paper as if it is the most natural thing to do. And this was in the *Journal of Physiology*. The same structure is found in the Michaelis-Menten paper, which was published (in German) in a biochemistry journal (Michaelis and Menten, 1913; Michaelis et al., 2011). If this was appropriate a century ago, why must details of mathematical models now be relegated to the back of papers (see, for example, Paulick et al. (2017), Ferreira et al. (2017), and Streichan et al. (2018))?

Many readers will appreciate that the issue I am raising about quantitative descriptions of living systems is closely associated with the tension that exists between the stereotypes of the biologist, who wants to incorporate all the complexity of a particular system, and the physicist who seeks generality and minimalism. As has been emphasized in other recent opinion pieces (Shou et al., 2015; Riveline and Kruse, 2017), the role of theory in biology has been growing and this development requires new ways of training scientists on both sides of the physics/biology divide. Less attention has been paid to providing concrete examples for the biology community of how physicists think about understanding data, and this essay’s goal, in part, is to address this lacuna.

Well aware of the risks of trying to speak for an entire community, below I take the reader through an example of how (at least some) physicists might go about describing a well-known phenomenon that shows up everywhere in biology – from the functioning of cellular receptors to bacterial chemotaxis, the propagation of action potentials, and fluorescence recovery after photobleaching (FRAP) experiments – namely, diffusion. Employing poetic license, I imagine that we are at a point in time when the diffusion equation itself was not known, nor was Fick’s Law, so both the experimental observations and theoretical analysis presented below are new and worthy of being described as Results.

I compose two versions of a Results section to indicate various ways of presenting the data and theory interleaved in a compact presentation that (I hope) is widely understandable by the community. The first version involves a ‘microscopic’ model that is a caricature of the biological system, but contains the essential ingredients to display the behavior observed on the large scale. The way in which microscopic parameters enter into the macroscopic answer turns out to be general (or, as physicists say, ‘universal’), a key take-home lesson. The second version – which is probably more challenging – involves the use of ‘dimensional analysis’, one of the most powerful methods of analyzing natural phenomena. Here, relationships between various quantities are deduced by examining the units in which they are measured (mass, length, time, charge, etc.). Introduced long ago, particularly in the work of Clerk-Maxwell, 1869, this technique can often lead to exact answers to problems, up to the proverbial ‘factors of two’.

## A discovery

**Figure 1.**
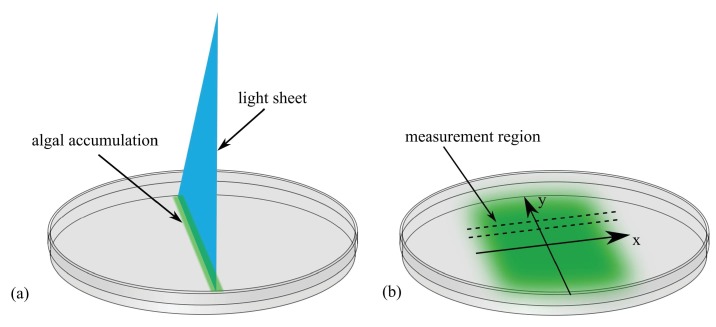
Experimental setup to study diffusion of the green alga *Chlamydomonas*. (**a**) A light sheet is used to gather the algae, which are swimming in a petri dish, into a narrow strip of cells along the y-axis. (**b**) After the light is turned off, the cells swim randomly and spread out. The concentration profile, C⁢(x,t), is then measured along a thin strip parallel to the x-axis; t is time.

Allow me to introduce our fictitious Professor Lamarr, who has been investigating how the single-cell green alga *Chlamydomonas* moves in response to light. She has discovered that if a narrow sheet of light is directed into an algal suspension in a petri dish (Figure 1a), the algae swim into the beam and form a concentrated line of cells. When the light is turned off and there is no more phototactic cue, the cells resume a random swimming motion described previously (Polin et al., 2009), in which every 10 seconds or so their roughly linear motion is interrupted by a turn: the angle of this turn falls within a distribution that has a mean of ~90 degrees. These random turns lead the population to spread out over time (Figure 1b). See 'Methods' for experimental details.

**Figure 2.**
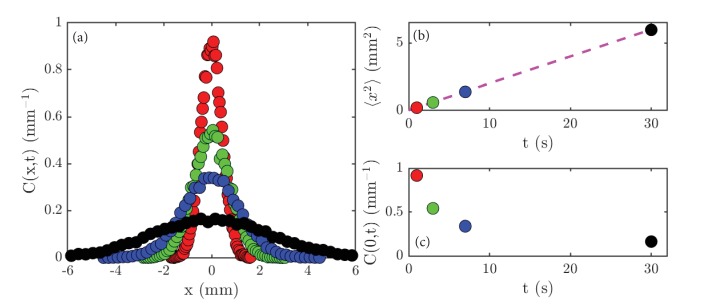
Experimental results on diffusion in a population of the green alga *Chlamydomonas*. (**a**) Concentration profiles, C⁢(x,t), normalized to unity, at the following times: 1 second (red), 3 seconds (green), 7 seconds (blue) and 30 seconds (black). (**b**) The variance, $\left\langle x^{2}\right\rangle$, of the data shown in (**a**) as a function of time; the dashed magenta line is a linear fit to the data. (**c**) The peak height, C⁢(0,t), of the data shown in (**a**) as a function of time.

Lamarr measures the normalized concentration profiles, C⁢(x,t), in a thin strip that is perpendicular to the initial line of cells, obtaining the data shown in Figure 2a. The sharply-peaked profile at early times gradually spreads out until the Petri dish is uniformly filled with cells. She measured the variance $\left\langle x^{2}\right\rangle$ of the concentration profile, and found the linear relation $\langle x^{2}\rangle =𝒟t$, with 𝒟=0.2 mm^2^/s (Figure 2b). Finally, the peak height C⁢(0,t) decays smoothly with time (Figure 2c). By systematic experimentation, she found that the basic results were insensitive to the precise size of the initial gathering, and that various swimming mutants of *Chlamydomonas* displayed the same behavior, albeit with different values of 𝒟.

### Results v1: Experimental observations explained by a microscopic model

**Figure 3.**
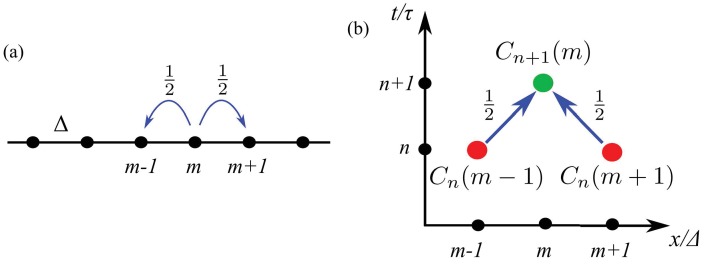
A random walk in one dimension. (**a**) A cell at site m moves with probability 1/2 to the left or right. (**b**) Diagram illustrating the counting that underlies the evolution equation (Equation 1).

In this version of Results, we begin with a theoretical model of the random motions of individual cells and deduce from it a population-level description with which to analyze the data. In the simplest picture, we assume that cells move only to the left and right along the x-axis, and the cells are constrained to sit on a discrete set of points, at positions $x_{m}=m\,\Delta$, where m=1,2,3,… (Figure 3a). Likewise, we assume time is discrete, so at each time $t_{n}=n\,\tau$, n=1,2,3,…, a cell moves with probability 1/2 to the left or right, as indicated by the arrows in Figure 3a.

In order to find an evolution equation for the probability $C_{n}\,\left(m\right)$ of finding a cell at position m⁢Δ⁢x at time n⁢Δ⁢t we observe (Figure 3b) that cells that appear at point m at time n+1 arrived there by moving to the right from point m-1 or by moving to the left from point m+1 at the previous time step (each with probability 1/2). Thus we can deduce that

(1) $$C_{n+1}\,\left(m\right)=\frac{1}{2}\,C_{n}\,\left(m+1\right)+\frac{1}{2}\,C_{n}\,\left(m-1\right).$$

We now imagine that these probabilities are varying sufficiently slowly in space and time that we can use the following Taylor expansions: $C_{n+1}\;(m)\;\;≃\;\;C_{n}\;(m)\;+\;\;\tau (\partial C_{n}(m)\;/\partial t)\;+\;\cdots$; and $C_{n}\;(m\;\pm \;\;1\;)\;\;≃$ $C_{n}(m)\pm \Delta (\partial C_{n}(m)/\partial x)+(\Delta^{2}/2)(\partial^{2}C_{n}(m)/\partial x^{2})+\cdots$. Collecting terms, we deduce that the ‘continuum limit’ for this one-dimensional random walk is

(2) $$\frac{\partial C}{\partial t}=D\frac{\partial^{2}C}{\partial x^{2}},\;with\;D=\frac{\Delta^{2}}{2\tau }.$$

We term this the ‘diffusion equation’, where the diffusion constant D has units of length${}^{2}$/time. Although the above was derived in the context of a model with discrete space and time coordinates, the crucial point is that we can more generally interpret Δ as the typical distance a cell travels between sharp turns, and τ as the time between such turns. If U is the swimming speed between turns, then Δ∼U⁢τ, so we can write $D=U^{2}\,\tau /2$. From tracking studies of *Chlamydomonas*, we know that U∼0.1 mm/s, and τ∼10 s, and therefore Δ∼1 mm and D∼0.1 mm${}^{2}$/s.

If we rewrite the diffusion equation (2) as ∂⁡C/∂⁡t=-(∂/∂⁡x)⁢(-D⁢∂⁡C/∂⁡x) then it can be written as

(3) $$\frac{\partial C}{\partial t}=-\frac{\partial J}{\partial x},\mathrm{where}J=-D\,\frac{\partial C}{\partial x},$$

where we identify the flux J as the number of cells passing through a given point x per unit time. This relationship implies that cells pass from regions of high concentration to regions of low concentration at a rate proportional the gradient of concentration. This ‘flux form’ of the diffusion equation guarantees that the total number of cells, $N=\int_{-\infty }^{\infty }𝑑x\,C\,\left(x,t\right)$, remains constant over time, since

(4) $$\begin{array}{ll} \frac{dN}{dt} & =\int_{-\infty }^{\infty }dx\frac{\partial C(x,t)}{\partial t}\\ & =-\int_{-\infty }^{\infty }dx\frac{\partial J}{\partial x}=J(-\infty )-J(+\infty ). \end{array}$$

Thus, provided the flux J goes to zero far away from our point of observation, N is constant.

**Figure 4.**
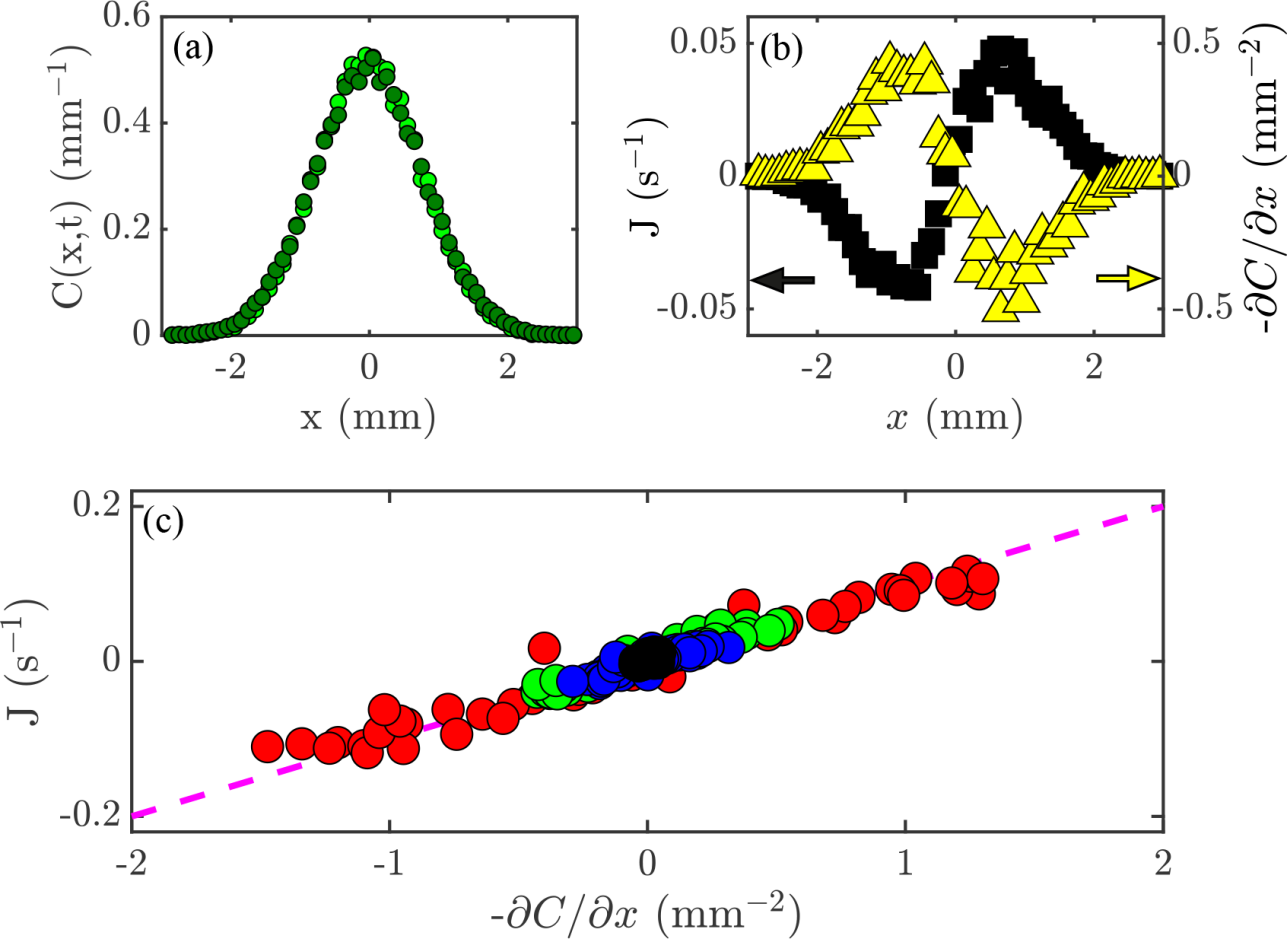
Flux and the diffusion equation. (**a**) Concentration profiles, C⁢(x,t), at times t=3 s 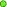
and t=3.2 s 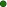. (**b**) The flux of cells past a given point, J (black; left axis), and the concentration gradient, ∂⁡C/∂⁡x (yellow; right axis), versus position, x. (**c**) Flux, J, versus concentration gradient, ∂⁡C/∂⁡x, for all the values of x and t shown in Figure 2a. The dashed magenta line has a slope D=0.1 mm^2^/s.

The relationship (Fick’s Law) J=-D⁢∂⁡C/∂⁡x can be tested experimentally. Lamarr recorded the distributions of cells at the times indicated in Figure 2 and then again 0.2 s later. As shown in Figure 4a for one pair, such measurements yield the flux, J, and concentration gradient, ∂⁡C/∂⁡x each as functions of x (Figure 4b), and we see that, apart from the overall scale, they are oppositely signed, as predicted by (3). But we can now go one step further and plot J at each point x and time t versus ∂⁡C/∂⁡x at those same x and t values. If the theory is correct, then every data set should collapse on to a single straight line, and indeed this is the case (Figure 4c). According to the theory above, the slope of the line in Figure 4c is the diffusion constant D; we obtain D=0.1 mm^2^/s, which is consistent with the microscopic interpretation in terms of motility.

### Results v2: Dimensional analysis leads to the diffusion equation

In this version of the Results section our goal is to infer directly from the data a differential equation for the time evolution of the algal concentration C⁢(x,t), which is measured in organisms per mm, hence units of 1/length. The variance $\left\langle x^{2}\right\rangle$ has, of course, units of length squared, so we can define a characteristic, time-dependent length $\ell \,\left(t\right)=\sqrt{\left\langle x^{2}\right\rangle }$. From the fit to the data in Figure 2b we infer that the width of C⁢(x,t) grows as

(5) $$\ell \,\left(t\right)∼\sqrt{𝒟\,t}.$$

A very natural question is whether ℓ⁢(t) is the only intrinsic length scale that can be extracted from the data. As C⁢(x,t) has units of number/length we can, without loss of generality, write $C\,\left(x,t\right)=\ell \,{\left(t\right)}^{-1}\,F\,\left(x,t\right)$ for some unknown function F that is itself dimensionless. And since F is dimensionless, it must be a function of a variable that is also dimensionless (similar to the way that sin⁡(θ) is a function of θ). Let us call this dimensionless variable ξ. With x and ℓ⁢(t) to work with, only the ratio is dimensionless, so we deduce that ξ=x/ℓ⁢(t). Thus, we expect

(6) $$C\,\left(x,t\right)=\frac{1}{\ell \,\left(t\right)}\,F\,\left(\frac{x}{\ell \,\left(t\right)}\right).$$

**Figure 5.**
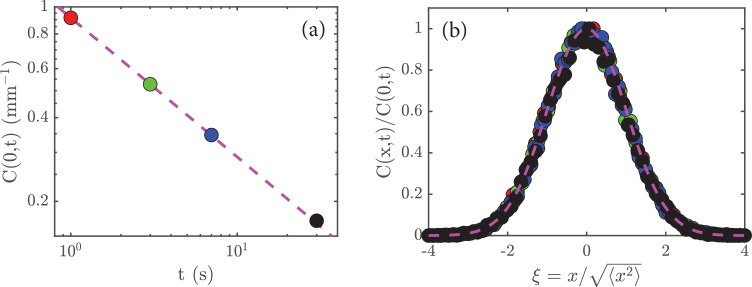
Rescaling the data. (**a**) The peak amplitude, C⁢(0,t), from Figure 2c plotted as a function time, t, on a log-log scale; the dashed magenta line has a slope of -1/2, which shows that $C\,\left(0,t\right)∼t^{-1/2}$. (**b**) When the data in Figure 2a are rescaled (see main text) and replotted, they collapse to a universal curve; the dashed magenta curve is the function $\exp\left(-\xi^{2}/2\right)$.

Let us now see if this form is consistent with the data. First, we note that it guarantees that the total number of cells, $N=\int_{-\infty }^{\infty }𝑑x\,C\,\left(x,t\right)$, does not change with time because

(7) $$\begin{array}{ll} N=\int_{-\infty }^{\infty }\;\;dx\;C(x,t) & =\int_{\infty }^{\infty }dx\frac{1}{\ell (t)}F\left(\frac{x}{\ell (t)}\right)\\ & \;\;\;\;\;=\int_{-\infty }^{\infty }\;d\xi F(\xi ), \end{array}$$

and $\int_{-\infty }^{\infty }𝑑\xi \,F\,\left(\xi \right)$ is a number that does not depend on time (just like $\int_{0}^{\pi }𝑑\theta \,\sin\left(\theta \right)$ is a number). Given (Equation 6), the peak concentration C⁢(0,t) is just F⁢(0)/ℓ⁢(t), where F⁢(0) is again just a number. With the scaling in (Equation 5) we deduce that $C\,\left(0,t\right)∼1/\sqrt{t}$. A replotting of the data in Figure 2c on a log-log scale shows that this is true (Figure 5a).

A significant prediction of the analysis leading to (6) is that the data at different times should collapse when plotted as C⁢(x,t)/C⁢(0,t) versus x/ℓ⁢(t), for this ratio is just F⁢(ξ)/F⁢(0). (Dividing C⁢(x,t) by C⁢(0,t) means that we rescale the heights of the various curves; and dividing x by ℓ⁢(t) means that we allow for expansion of the initial concentration of cells). If this holds, then it implies that ℓ⁢(t) is the only characteristic length in the system. A test of this is shown in Figure 5b, where we see a good collapse of the data to a universal curve.

It is natural to seek a differential equation that is consistent with the scaling $x^{2}∼t$ and would provide a quantitative prediction of the function F. First we consider if inertia is relevant in this system. We know from fluid dynamics that inertia is irrelevant when the Reynolds number R⁢e=U⁢L/ν is much less than unity: U is the typical speed of a particle, L is the typical length of a particle, and ν is the kinematic viscosity (which is defined as ν=η/ρ, where η is the fluid viscosity and ρ is the fluid density). For *Chlamydomonas* swimming in water ($U∼10^{-2}$ cm/s, $L∼10^{-3}$ cm, and $\nu =10^{-2}$ cm^2^/s), we have $R\,e∼10^{-3}$ and inertia is indeed negligible.

The differential equation we seek will have derivatives both in time and in space. In the absence of inertia, we expect that the equation for C⁢(x,t) should only involve first-order derivatives in time (as second derivatives would imply inertia and accelerations). With the scaling $x^{2}∼t$ we expect two space derivatives for one time derivative, so a consistent equation would be

(8) $$\frac{\partial C}{\partial t}=D\,\frac{\partial^{2}C}{\partial^{2}x},$$

where the parameter D should be proportional to the empirical 𝒟 obtained from Figure 2b.

To find a solution of (Equation 8) in the form of (Equation 6), we use D to construct a length $l=\sqrt{D\,t}$ and find (see *Mathematical Details*) the normalized distribution

(9) $$C\,\left(x,t\right)=\frac{1}{\sqrt{4\,\pi \,D\,t}}\,\exp\left(-\frac{x^{2}}{4\,D\,t}\right).$$

Given this distribution, we compute the variance as

(10) $$\left\langle x^{2}\right\rangle =\int_{-\infty }^{\infty }x^{2}\,C\,\left(x,t\right)=2\,D\,t.$$

Comparing with our empirical observation (Equation 5), we deduce 𝒟=2D (the promised factor of two!) and therefore that the dimensionless function is $F\,\left(\xi \right)={\left(2\,\pi \right)}^{-1/2}\,\exp\left(-\xi^{2}/2\right)$. The ratio $F\,\left(\xi \right)/F\,\left(0\right)=\exp\left(-\xi^{2}/2\right)$ is shown as the dashed line in Figure 5b, in good agreement with the data.

Taken together, the experimental observations in Figure 2 and the phenomenological analysis above, confirmed in Figure 5, suggest that the diffusion equation in (Equation 8) provides a sound description of the spreading of cells that execute random motions. It indicates that different organisms, with different diffusion constants, obey the same fundamental scaling laws, insensitive to the details of the underlying random motions. Note that at this level of analysis we do not have a microscopic *interpretation* of the diffusion constant in terms of the fluid viscosity and aspects of cell motility; it is simply a phenomenological parameter that can be used to characterize a given microorganism. On the other hand, if we knew from microscopical observations that an organism’s motion consists of straight segments interrupted by random reorientations, as in the case of *Chlamydomonas* and indeed *E. coli* (Berg, 1993), then by dimensional analysis (again) we could deduce $D∼\Delta^{2}/\tau ∼U^{2}\,\tau$ in terms of the run length Δ, speed U, and time between turns τ.

## Discussion

I have presented two ways of interleaving data and theory in a Results section as a way of indicating how quantitative principles can be used to derive new insight into phenomena. In one, a microscopic model led directly to the diffusion equation, whose structure led to the ‘rediscovery’ of Fick’s law, which was confirmed from the data. In the second, the principles of dimensional analysis and some phenomenological reasoning led us to postulate a ‘new’ diffusion equation as a concise encoding of the experimental observations. Each of these approaches used nothing more than basic algebraic manipulations and elementary differential equations.

Returning to the referees who spoke of inferences drawn directly from the data, I would ask: “What language does the data speak?” The answer would appear to depend on one’s background. The inferences I drew from Lamarr’s data were based on experience with understanding continuum and nonequilibrium phenomena, subjects which are less common in the undergraduate physics curriculum than one would hope, and very seldomly found in biology curricula. So, I would indeed advocate a more holistic education for both biologists and physicists (Goldstein et al., 2005).

It might be argued that the particular example I presented here is unusual, but in fact these very same considerations (dimensional analysis, scaling collapse of data, etc.) are to be found in many other places in biophysics. Excellent examples are work on metabolic scaling laws (West et al., 1997) and on stem cell replacement dynamics (Lopez-Garcia et al., 2010).

More importantly, I am not trying to emphasize any particular method in the physicist’s toolbox, but rather a mindset that is about model-building *and testing* as part of the results presented to the reader. This mindset is particularly relevant when the theory is formulated first and the experiment is undertaken to test it. But even when the experiment comes first there may be a need to use theory as a sanity check on one’s observations (Meister, 2016). This also brings us to the delicate issue of the extent to which research should actually be ‘hypothesis driven’, as discussed provocatively by Milner, 2018: I will leave that Pandora’s box closed for the moment.

Finally, one could argue that the diffusion equation is ‘just a model’ or ‘just a theory’ and should, therefore, not be considered as a Result because, unlike the data, it could be shown to be incorrect. With my experimentalist hat on, I find that argument weak: almost every experiment has potentially confounding aspects, and despite our best efforts to control them, these effects can produce spurious results. After all, how many hundreds or thousands of papers must have been written about stomach ulcers before Marshall and Warren, 1984 discovered that *H. pylori* was so often the culprit? So, while it is certainly the case that many of the models discussed in biology papers do not have the status of fundamental laws, I think that it is contrary to the scientific method to view the fact that they may be superseded as a weakness. If theories are crafted the right way they have utility even if proven wrong, sometimes especially if proven wrong!

This essay has touched on two tensions – between theory and experiment, and between the cultures of physics and biology. The differences between the cultures have implications not only for how data is interpreted, but also for what qualifies as “interesting” and who gets to frame the questions: an enlightening debate on this issue was aired more than 20 years ago by Adrian Parsegian and Robert Austin (Parsegian, 1997; Huebner et al., 1997). For example, it might be argued that biologists may not really be interested in the fact that a new equation has been derived that provides an approximate description of a given system, and this could be a reason not to publish a theoretical work in a biology journal. The example I provide here shows how this need not be an empty exercise, but can lead to testable, mechanistic predictions such as the relationship between flux and concentration gradient (Fick’s Law, rediscovered). One need only consult the seminal work of Turing (1952) on biological pattern formation or of Hodgkin and Huxley (1952) on action potentials to see the importance of having a mathematical encoding of diffusion to study its mechanistic implications. Likewise, a physics-oriented experimental paper, even one that deals with living organisms, may also not be seen as interesting to biologists because the questions appear unfamiliar. For truly interdisciplinary journals, easing this tension is perhaps the greatest challenge.

## Methods

### Generating the data

Full disclosure – rather than do the experiments, I numerically solved the Langevin equation d⁢x/d⁢t=η⁢(t) for the time evolution of the position x⁢(t) for a single alga undergoing random motion, where η⁢(t) is a random variable with zero mean and temporal correlation function $\left\langle \eta \,\left(t\right)\,\eta \,\left(t^{\prime }\right)\right\rangle =2\,D\,\delta \,\left(t-t^{\prime }\right)$. In the results described here, I set D=0.1 mm^2^/s, approximately that of *Chlamydomonas* (Polin et al., 2009). The equation was integrated forward a time increment δ⁢t from time index i to i+1 using the discrete representation $x_{i+1}=x_{i}+\sqrt{2\,D\,\delta \,t}\,\eta_{i}$, where $\eta_{i}$ is a normally distributed random variable. The data represent averages over 30,000 realizations.

### Mathematical details

To obtain the normalized concentration profile (Equation 9) we simply substitute the latter into the diffusion (Equation 8), with $\chi =x/\sqrt{D\,t}$. We obtain

(11) $$\frac{d^{2}\,F}{d\,\chi^{2}}+\frac{1}{2}\,\left(F+\chi \,\frac{d\,F}{d\,\chi }\right)=0.$$

Integrating (Equation 11) once and imposing the boundary condition that F→0 as χ→∞ we obtain d⁢F/d⁢χ+(1/2)⁢χ⁢F=0, which integrates to

(12) $$F(\xi )=A\exp(-\chi^{2}/4).$$

Normalizing the associated concentration profile and re-expressing the result in terms of the original variables yields the result (Equation 9).
